# Integrated driver mutations profile of chinese gastrointestinal-natural killer/T-cell lymphoma

**DOI:** 10.3389/fonc.2022.976762

**Published:** 2022-08-18

**Authors:** Shanshan Li, Tingzhi Liu, Hailing Liu, Xiaohui Zhai, Taiyuan Cao, Hongen Yu, Wanjia Hong, Xiaoru Lin, Ming Li, Yan Huang, Jian Xiao

**Affiliations:** ^1^ Department of Medical Oncology, The Sixth Affiliated Hospital of Sun-Yat Sen University, Guangzhou, China; ^2^ Guangdong Provincial Key Laboratory of Colorectal and Pelvic Floor Diseases, The Sixth Affiliated Hospital, Sun Yat-sen University, Guangzhou, China; ^3^ Department of Medical Hematology, The Sixth Affiliated Hospital of Sun Yat-Sen University, Guangzhou, China; ^4^ Department of Pathology, The Sixth Affiliated Hospital of Sun-Yat Sen University, Guangzhou, China

**Keywords:** driver gene, mutation, gastrointestinal-natural killer/T-cell lymphoma (GI-NKTCL), immune infiltration, genomic analysis

## Abstract

**Background:**

One of the most common nasal external sites in extranodal Natural Killer/T-cell lymphoma (NKTCL) is in the gastrointestinal (GI) system. Despite this, reports on gastrointestinal-Natural Killer/T-cell lymphoma (GI-NKTCL) are very few. To obtain a better understanding of this manifestation of NKTCL, we conducted a retrospective study on GI-NKTCL to analyze its clinical features, genomic changes and immune infiltration.

**Methods:**

We retrospectively collected patients diagnosed with GI-NKTCL in the Sixth Affiliated Hospital of Sun Yat-sen University from 2010 to 2020. From this cohort we obtained mutation data *via* whole exome sequencing.

**Results:**

Genomic analysis from 15 patients with GI-NKTCL showed that the most common driving mutations were ARID1B(14%, 2/15), ERBB3(14%, 2/15), POT1(14%, 2/15), and TP53(14%, 2/15). In addition, we found the most common gene mutation in patients with GI-NKTCL to be RETSAT(29%, 4/15) and SNRNP70(21%, 3/15), and the most common hallmark pathway mutations to be G2M checkpoint pathway (10/15, 66.7%), E2F targets (8/15, 53.3%), estrogen response late (7/15, 46.7%), estrogen response early (7/15, 46.7%), apoptosis (7/15, 46.7%) and TNFA signaling *via* NFKB (7/15, 46.7%). In the ICIs-Miao cohort, SNRNP7-wild-type (WT) melanoma patients had significantly prolonged overall survival (OS) time compared with SNRNP7 mutant type (MT) melanoma patients. In the TCGA-UCEC cohort, the patients with RETSAT-MT or SNRNP7-MT had significantly increased expression of immune checkpoint molecules and upregulation of inflammatory immune cells.

**Conclusions:**

In this study, we explored GI-NKTCL by means of genomic analysis, and identified the most common mutant genes (RETSAT and SNRNP70), pathway mutations (G2M checkpoint and E2F targets) in GI-NKTCL patients. Also, we explored the association between the common mutant genes and immune infiltration. Our aim is that our exploration of these genomic changes will aid in the discovery of new biomarkers and therapeutic targets for those with GI-NKTCL, and finally provide a theoretical basis for improving the treatment and prognosis of patients with GI-NKTCL.

## Introduction

Extranodal natural killer/T-cell lymphoma (ENKTCL) is a rare type of Non-Hodgkin’s Lymphoma (NHL) (1–3). It is characterized by extranodal involvement, with tumor cells predominantly from peripheral mature NK cells, and less from cytotoxic T cells. One site of extranodal involvement is the gastrointestinal system (GI-NKTCL); however, reports of patients with primary GI-NKTCL are rare (4, 5). Currently, the treatment of NKTCL is still based on radiotherapy, or a combination of radiotherapy and chemotherapy, and stage I/II NKTCL patients showed locoregional control rates of >90% and five-year overall survival (OS) rates of approximately 70–90% (6). Stage III/IV NKTCL patients were treated with combination chemotherapy that includes menadione enzymes (7–9). The SMILE protocol (dexamethasone, methotrexate, ifosfamide, l-asparaginase, and etoposide) can be the standard of care for stage III/IV NKTCL patients, with one-year OS rates of >50%.

Whole exome sequencing (WES), also known as targeted exon capture, is a new technology which explores disease-related genes by studying only the coding regions of the human genome (10–13). It is comparable to whole genome sequencing (WGS), which samples the entire human genome. WES, however, only samples 1%, while still allowing for analysis of single nucleotide variants (SNV), insertion-deletions (indel), and structural variations (SV) of pathogenic genes (14, 15). Applying this new generation sequencing technology to ENKTCL further confirmed the complexity of gene changes present, including the deletion of chromosome 6q, and PRDM1, ATG5, AIM1, FOXO3, HACE1 as common deletion segments on chromosome 6q (16). Additionally, tumor-related genes with known mutations include some tumor suppressor genes (such as TP53, DDX3X, and MGA), JAK/STAT pathway genes (such as JAK3, STAT3, and STAT5), and some epigenetic modified genes (such as KMT2D, BCOR, ARID1A, and EP300) (4, 17–20). The proteins encoded by these mutant genes show a loss of normal original functions, with some increase of invasive functions. Additionally, the tumor immune microenvironment (TIME) plays important role in the tumor development and progression. Although some genetic studies on ENKTCL have been conducted, genomic changes and immune infiltration in GI-NKTCL have not been fully explored due to the low number of reported cases.

In this study, we carried out WES on patients diagnosed with GI-NKTCL in China, explored the genomic-level changes of these patients, and analyzed the mutational profiles and common pathway mutation landscapes. Also, we analyzed the relationship between the top mutated genes and the prognosis of the immune checkpoint inhibitors (ICIs) treatment, and TIME. We hope that by deeply studying the molecular mechanism of the pathogenesis of GI-NKTCL, looking specifically for potential molecular targets and exploring new treatment methods, we can improve the prognosis of patients with GI-NKTCL.

## Methods

### Sample collection and raw data sequencing of NK/T cell lymphoma

We retrospectively collected 15 samples of NKTCL from patients diagnosed with GI-NKTCL at the Sixth Affiliated Hospital of Sun Yat-sen University from 2010 to 2020. The pathological diagnosis of GI-NKTCL was based on the revised European and American lymphoma (REAL) classification, and the classification standard of the World Health Organization (WHO). The study was approved by the Research Ethics Committee of the Sixth Affiliated Hospital of Sun Yat-sen University. The details of the raw data sequencing are listed in the Supplementary Methods. Additionally, we downloaded the WES data from the NKTCL cohort reported by Li et al. (21).

### Pan-cancer datasets and ICIs datasets collection

Data sets for the following 33 cancer types were downloaded from The Cancer Genome Atlas (TCGA) database: adrenocortical carcinoma (ACC), bladder urothelial carcinoma (BLCA), breast invasive carcinoma (BRCA), cervical squamous cell carcinoma and endocervical adenocarcinoma (CESC), cholangiocarcinoma (CHOL), colon adenocarcinoma (COAD), colorectal cancer (CRC), lymphoid neoplasm diffuse large B-cell lymphoma (DLBC), esophageal carcinoma (ESCA), glioblastoma multiforme (GBM), head and neck squamous cell carcinoma (HNSC), kidney chromophobe (KICH), kidney renal clear cell carcinoma (KIRC), kidney renal papillary cell carcinoma (KIRP), acute myeloid leukemia (LAML), brain lower grade glioma (LGG), liver hepatocellular carcinoma (LIHC), lung adenocarcinoma (LUAD), lung squamous cell carcinoma (LUSC), mesothelioma (MESO), non-small cell lung cancer (NSCLC), ovarian serous cystadenocarcinoma (OV), pancreatic adenocarcinoma (PAAD), pheochromocytoma and paraganglioma (PCPG), prostate adenocarcinoma (PRAD), rectum adenocarcinoma (READ), sarcoma (SARC), skin cutaneous melanoma (SKCM), stomach adenocarcinoma (STAD), testicular germ cell tumors (TGCT), thyroid carcinoma (THCA), thymoma (THYM), uterine corpus endometrial carcinoma (UCEC), uterine carcinosarcoma (UCS), and uveal melanoma (UVM) (22). Also, we downloaded a pan-cancer data set published by Zehir et al. from the cBioPortal web tool (https://www.cbioportal.org/) (23). We downloaded the ICIs-cohorts reported by the Miao et al. (24). and Allen et al. (25). to explore the association between the top mutated genes and ICIs-related prognosis.

### Genome analysis

The somatic mutation data of our cohort was subjected to preprocessing and any synonymous mutations were deleted. Based on the non-synonymous mutation data, we identified the types and frequency of gene mutations in GI-NKTCL that had a mutation frequency greater than or equal to 2. In addition, we downloaded the Hallmark pathway gene set (h.all.v7.1.symbols.gmt) from the MsigDB database (http://www.gsea-msigdb.org/gsea/downloads.jsp) and used this to count the number of gene mutations in different Hallmark pathways in each patient. We calculated the number of gene mutations in different Hallmark pathways for each cancer type in both the TCGA-Pancancer and Zehir-Pancancer cohort and, using the MutationMapper function in the cBioPortal web tool (https://www.cbioportal.org/mutation_mapper), visualized the mutation sites of the two genes with the highest frequency of mutation in GI-NKTCL. Additionally, we counted the proportion of six single base substitution types in our GI-NKTCL cohort.

### Immune infiltration

The gene lists of the immune checkpoint molecules were downloaded from the previous study (25). The proportion of the immune cells was estimated by CIBERSORT (25).

### Statistical analysis

Fisher’s exact test was used to analyze the mutual exclusion and co-occurrence of gene mutations. Heatmaps were visualized using the ComplexHeatmap (26) and pheatmap (27) R packages, and histograms were visualized using the ggplot2 (28) R package. The Maftools (29) R package was used for mutual exclusion and co-occurrence between gene mutations. All analyses in this study were carried out using R software (Version 3.8.1). A P value of less than 0.05 was regarded as statistically significant, and the P value was bilateral.

## Results

### Overview of the mutational spectrum and pattern of GI-NKTCL

**Figure 1:**
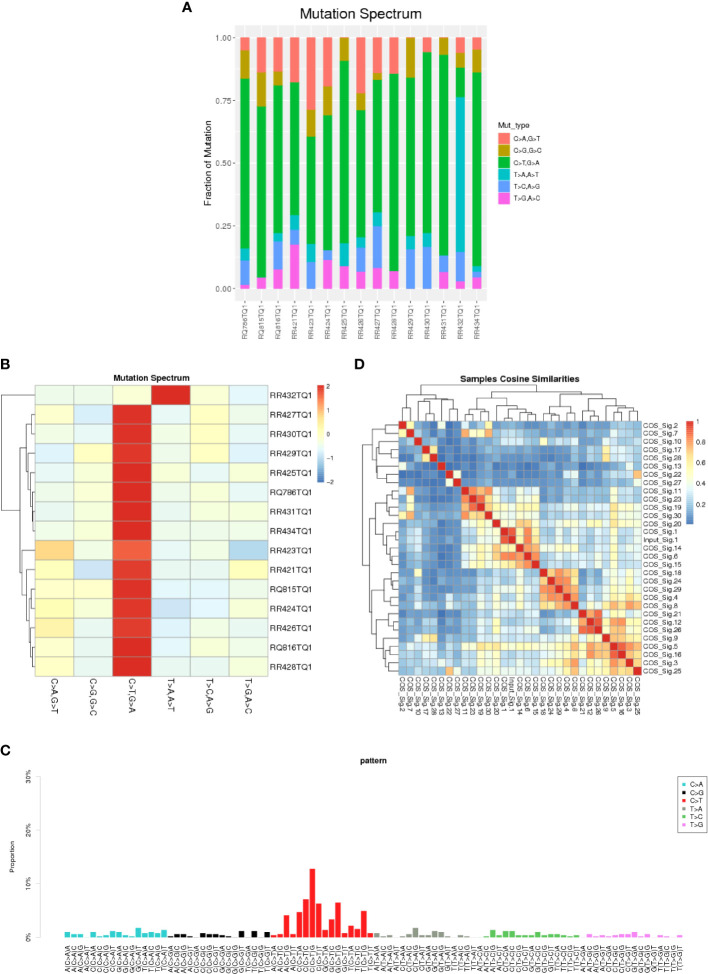
A barplot **(A)** and heatmap **(B)** depicting the mutation spectrum of the GI-NKTCL cohort. **(C)** The mutation pattern of the GI-NKTCL cohort obtained *via* Nonnegative Matrix Factorization (NMF). **(D)** Heatmap showing the correlation between the COSMIC signatures.

In this study, we collected data from 12 male and 3 female patients with GI-NKTCL, with an average age of 49.6 years old (comparable to the general characteristics reported by ENKTL) (30). The basic clinical features of our cohort are shown in **Table 1**. Of the primary tumor sites, six cases were colonic (40.0%), four cases were ileocecal (26.7%), and five cases were small intestinal (33.3%). Five cases were treated with surgery (33.3%), and ten cases were treated with combined surgery and chemotherapy (66.7%). One patient survived (6.7%), and the remaining 11 patients died (93.3%). **Supplementary Figure 1** shows the overall survival (OS) time of GI-NKTCL. Also, We calculated and counted the proportion of six single base substitution types for the SNV detection results of all samples, and found that C>T/G>A accounted for the most single base substitution types in each patient (**Figure 1A**), which was analogous to the somatic SNV spectrum in other cancers (including B-cell acute lymphoblastic leukemia and NKTCL) (31, 32). On the other hand, T>A/A>T had the least proportion of single base substitution types in each patient. Clustering the samples according to the distribution of mutation types resulted in the cluster heat map seen in **Figure 1B**, representing the mutation spectrum in the cohort. A variety of mutation processes, such as mismatching in the process of DNA replication, induction of endogenous or exogenous mutagens, and defects of DNA repair mechanism, were responsible for producing somatic mutations. These processes give rise to specific combinations of mutation types, which can be seen as mutation patterns. Point mutations can be divided into 96 types by considering the base types at the 1bp positions upstream and downstream of the point mutation site. The mutation pattern of the 96 mutation types in all samples is presented in **Figure 1C**. Based on cosine similarity, we clustered the mutation pattern with 30 known mutation features in the COSMIC website (https://cancer.sanger.ac.uk/signatures/signatures_v2/). We found the mutation pattern of GI-NKTCL was highly correlated with COSMIC signature 1 (cosine similarity score: 0.91) (**Figure 1D**). COSMIC signature 1 was the result of an endogenous mutational process initiated by spontaneous deamination of 5-methylcytosine and associated the age of cancer diagnosis (33).

**Table 1:** Clinical features of GI-NKTCL cases (N = 15).

Characteristics	N
Age	
≤60	10
>60	5
Sex	
Female	3
Male	12
Primary Site	
Rectum	5
Colon	6
Small intestine	4
LDH Status	
Increase	6
Normal	9
CD56	
(+)	7
(-)	7
Missing	1
Surgery	
YES	15
NO	0
Treatment	
Surgery	10
Surgery+Chemotherapy	5
Survival status	
Dead	14
Alive	1

### Panoramic overview of mutations in GI-NKTCL

**Figure 2:**
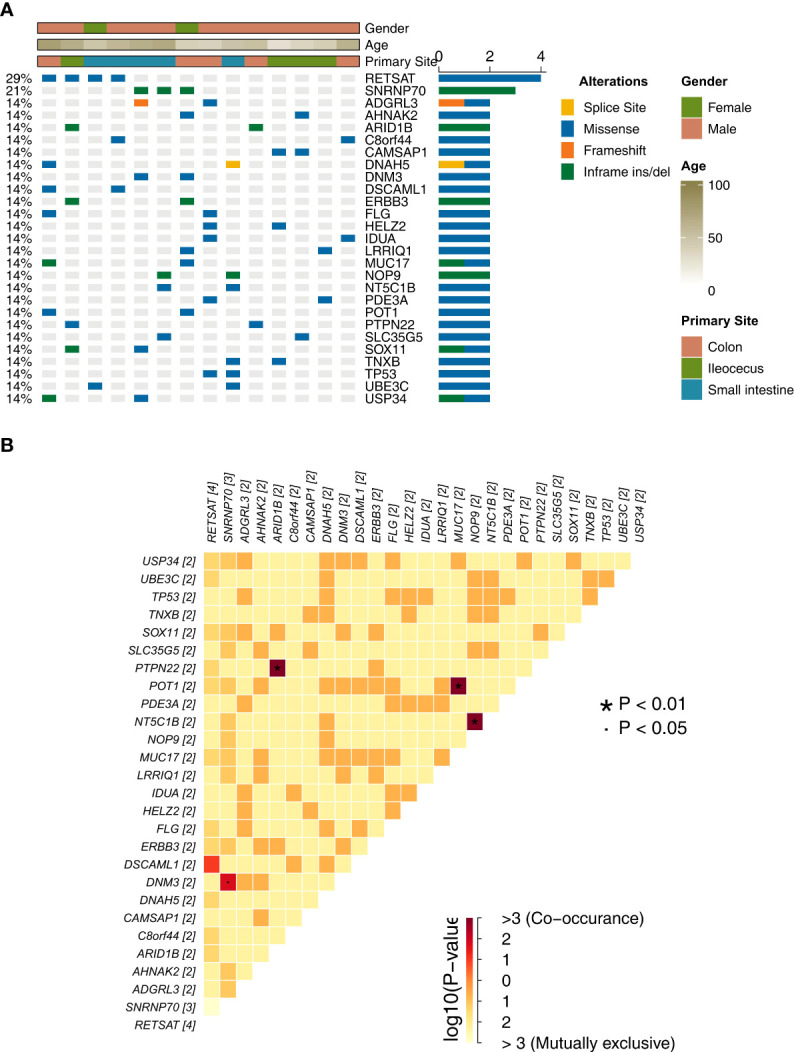
**(A)** The top 27 mutations of the GI-NKTCL cohort. The clinical feature of each patient is annotated in the top panel. **(B)** The co-occurrence and mutually exclusivity of the top 27 mutations (shown in Panel **A**).

**Figure 3:**
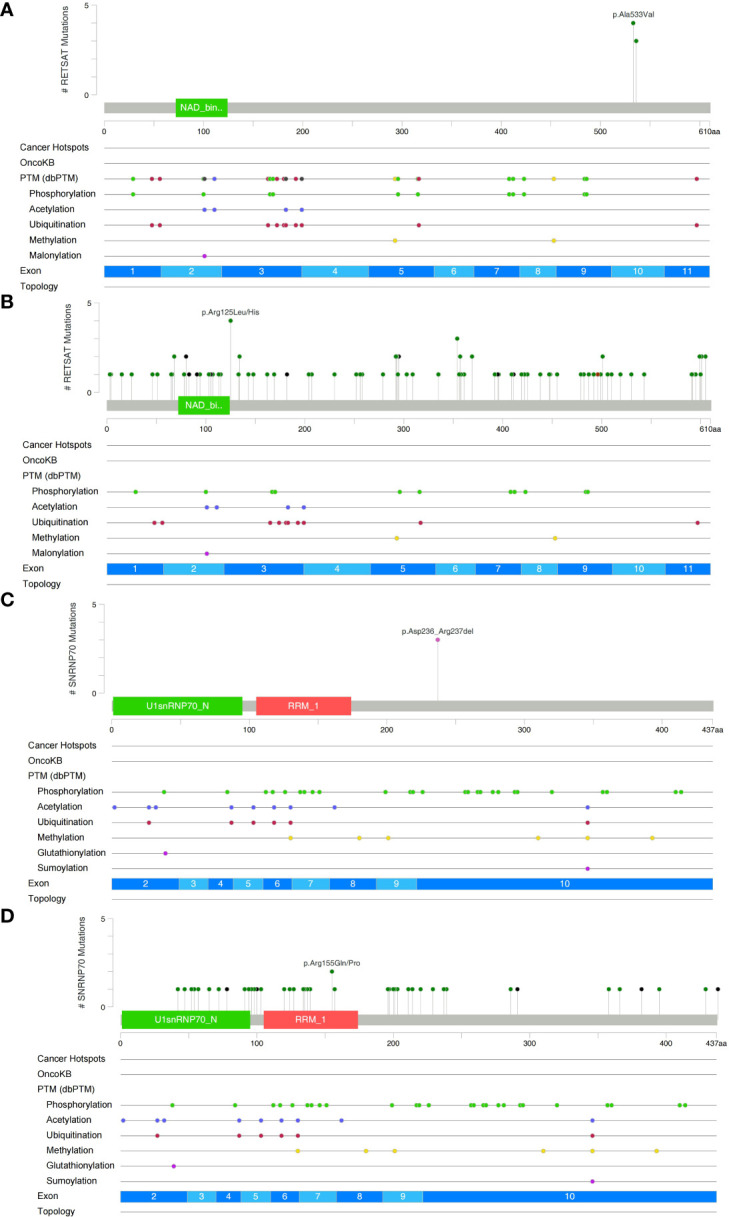
Lollipop plot illustrating RETSAT mutations in the GI-NKTCL **(A)** and TCGA-Pancancer **(B)** cohorts. Lollipop plot illustrating SNRNP70 mutations in the GI-NKTCL **(C)** and TCGA-Pancancer **(D)** cohorts.

We analyzed and visualized the data of non-synonymous somatic mutations from patients diagnosed with GI-NKTCL. The genes with the highest mutation frequency in these patients were identified as RETSAT (29%, 4/15); SNRNP70 (21%, 3/15); and ADGRL3, AHNAK2, ARID1B, C8orf44, CAMSAP1, DNAH5, DNM3, DSCAML1, ERBB3, FLG, HELZ2, IDUA, LRRIQ1, MUC17, NOP9, NT5C1B, PDE3A, POT1, PTPN22, SLC35G5, SOX11, TNXB, TP53, UBE3C, and USP34 (each being 14%, 2/15) (**Figure 2A**). Additionally, the main type of gene mutation was found to be missense, followed by inframe ins/del, splice site, and finally frameshift mutation. Following this, we analyzed the mutual exclusion and co-occurrence of the gene mutations with the highest mutation frequency. As can be seen in **Figure 2B**: mutations in MUC17 gene and POT1 gene often occur together (P < 0.05); mutations in NT5C1B gene and NOP9 gene usually occur together (P < 0.05); and mutations in PTPN22 gene and ARID1B gene will occur at the same time (P < 0.05). The protein structural mutation points in the two genes with the highest mutation frequency (RETSAT and SNRNP70) in patients with GI-NKTCL were visualized in the form of lollipop plots. In the GI-NKTCL cohort, we found the protein mutation site of RETSAT to be mainly p.Ala533Val (**Figure 3A**), while in the TCGA-Pancancer cohort, it is mainly p.Arg125Leu/His (**Figure 3B**). In the GI-NKTCL cohort, we found the protein structural mutation site of SNRNP70 to be mainly p.Asp236_Arg237del (**Figure 3C**), and in TCGA-Pancancer cohort, it is mainly p.Arg155Gln/Pro, occurring predominantly in the RRM_1 domain (**Figure 3D**). The genes with the highest mutation frequencies in NKTCL (Li-cohort) are shown in **Supplementary Figure 2**. The genes with the highest mutation frequency in these patients were identified as FSIP2 (22%), GNAQ (22%), USP8 (22%), IGFN1 (19%), KMT2D (19%), LOC401052 (19%), MUC21 (19%) and TTN (19%) (**Supplementary Figure 2**).

### Overview of abrupt change of Hallmark pathway signaling in GI-NKTCL

**Figure 4:**
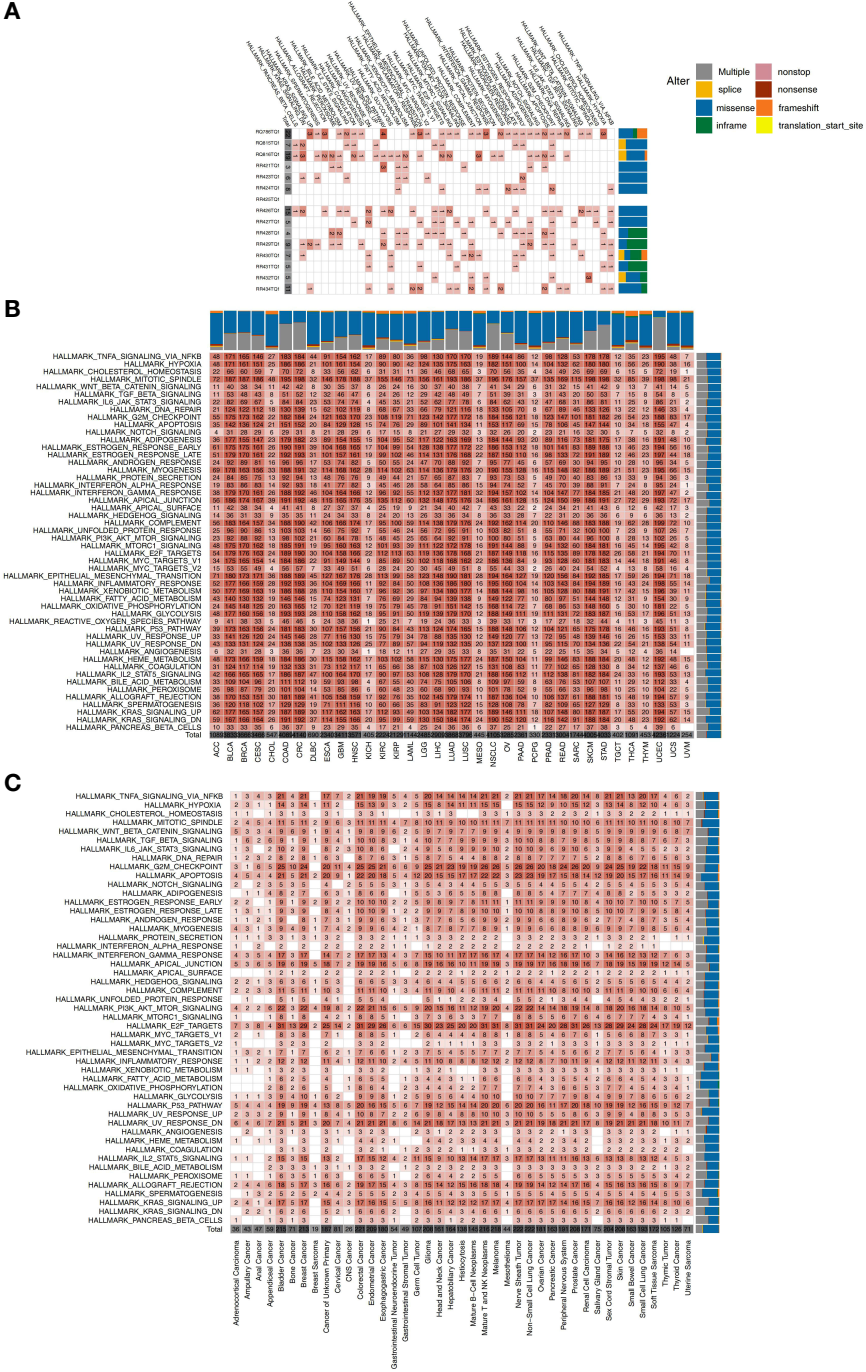
**(A)** The mutation counts of hallmark signaling pathways for each patient in the GI-NKTCL cohort. The mutation counts of hallmark signaling pathways for each cancer type in the TCGA-Pancancer **(B)** and Zehir-Pancancer **(C)** cohorts.

Hallmark pathways are landmark gene sets which can represent biological processes and states and may also play a vital role in tumor development. Therefore, we explored the mutations of each GI-NKTCL patient in a variety of hallmark pathways (**Figure 4A**). We found mutations in the G2M checkpoint pathway 66.7% of patients (10/15). The next most common mutated pathways were E2F targets (8/15, 53.3%), estrogen response late (7/15, 46.7%), estrogen response early (7/15, 46.7%), apoptosis (7/15, 46.7%), and TNFA signaling *via* NFKB (7/15, 46.7%). The mutation of various cancer types in different hallmark pathways in the TCGA-Pancancer and Zehir-Pancancer cohorts are shown in **Figures 4B, C** respectively. Found in all samples (41/41, 100%) were mutations in apoptosis, E2F targets, mitotic spindle, myogenesis, p53 pathway, PI3K-AKT-mTOR signaling, TGF beta signaling, UV response DN, UV response UP, and WNT beta catenin signaling.

### Association between most frequently mutated genes (RETSAT and SNRNP70) and immune infiltration

**Figure 5:**
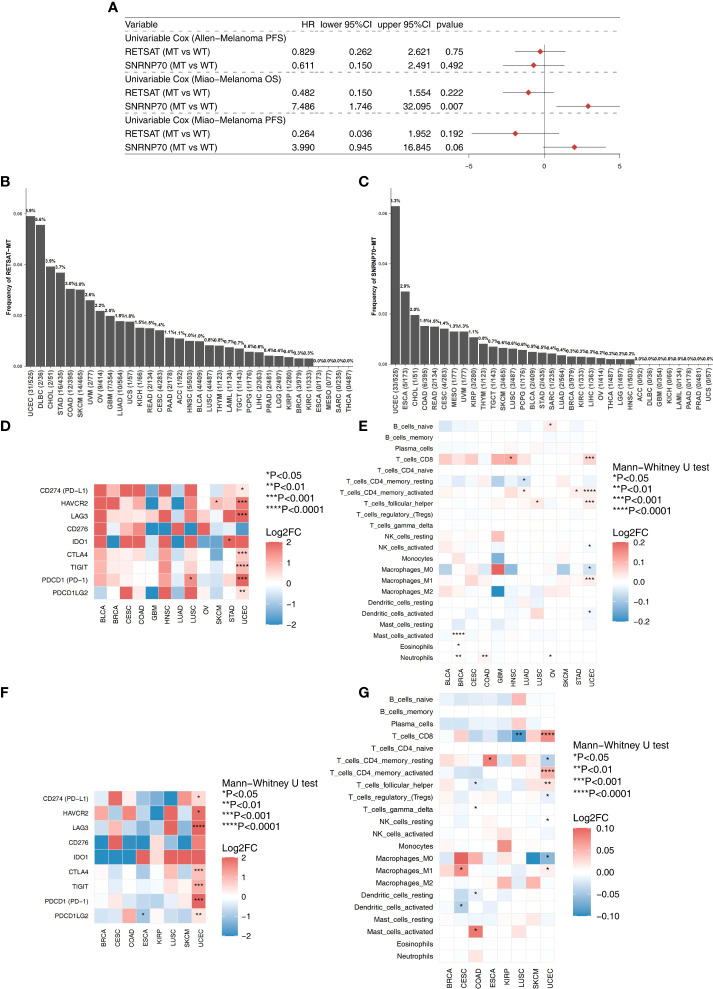
**(A)** The univariable cox regression model of the top mutated genes (RETSAT and SNRNP70) in the two ICIs-treated cohorts (Allen-Melanoma and Miao-Melanoma). **(B)** The mutation frequencies of the RETSAT in the 33 cancer types of TCGA database. **(C)** The mutation frequencies of the SNRNP70 in the 33 cancer types of TCGA database. **(D)** Heatmap depicted the logFC of the expression levels of the immune checkpoint molecules between the RETSAT-MT and RETSAT-WT among multiple cancer types (TCGA database). **(E)** Heatmap depicted the logFC of the immune cells scores estimated by the CIBERSORT method between the RETSAT-MT and RETSAT-WT among multiple cancer types (TCGA database). **(F)** Heatmap depicted the logFC of the expression levels of the immune checkpoint molecules between the SNRNP70-MT and SNRNP70-WT among multiple cancer types (TCGA database). **(G)** Heatmap depicted the logFC of the immune cells scores estimated by the CIBERSORT method between the SNRNP70-MT and SNRNP70-WT among multiple cancer types (TCGA database).

We explored the effect of the most frequently mutated genes (RETSAT and SNRNP70) on the prognosis of the two ICI-treated cohorts (Allen-Melanoma and Miao-Melanoma). We found that SNRNP70-MT melanoma had a significantly decreased OS time compared to SNRNP70-WT melanoma (**Figure 5A**). In the TCGA cohort, we found that RETSAT was the most frequently mutated gene in the UCEC (5.9%,31/525), DLBC (5.6%,2/36), CHOL (3.9%,2/51), STAD (3.7%,16/435), COAD (3.0%,12/395), and SKCM (3.0%,14/465) subtypes (**Figure 5B**). **Figure 5C** shows that SNRNP70 was the most frequently mutated gene in the UCEC (6.3%,33/525), ESCA (2.9%,5/173), CHOL (2.0%,1/51), COAD (1.5%,6/395), and READ (1.5%,2/134). In the TCGA-UCEC cohort, RETSAT-MT was associated with higher expression of immune checkpoint molecules (including PD-L1, HAVCR2, LAG3, CTLA4, TIGIT, PD-1, and PDCD1LG2; **Figure 5D**) and immune cell enrichment (including CD8+ T cells, activated memory CD4+ T cells, and M1-macrophages; **Figure 5E**). Similarly, we found that SNRNP70-MT cases in the TCGA-UCEC cohort had increased expression of immune checkpoint-related genes (**Figure 5F**). Compared with SNRNP70-WT, SNRNP70-MT UCEC patients had significant enrichment of CD8+ T cells, activated memory CD4+ T cells, follicular helper T cells, and M1-macrophages (**Figure 5G**).

## Discussion

The prevalence of ENKTCL is highest in Asian and Latin American countries. Primary GI-NKTCL is extremely rare (1–3), with a limited number of reported cases and very few studies specifically focused on this subtype of NKTCL (34–37). Due to the rarity of the disease, the clinical, genomic and immune characteristics have not yet been clarified. Thus, we carried out this study to explore the genomic changes, mutation panorama, and common pathway mutations present, utilizing WES data from 15 Chinese GI-NKTCL patients. We have provided a comprehensive description of the genomic features of GI-NKTCL through bioinformatics, such as gene mutation patterns (base pair alterations, COSMIC signature), high-frequency mutations, and common pathology-related pathways with high-frequency mutations. Additionally, we explored the relationship between the high-frequency mutations and ICIs-related prognosis, and immune infiltration (including immune checkpoint inhibitors and immune cells). The discovery of commonly mutated genes and signaling pathways can offer salient theoretical guidance for the prevention and treatment of future cancer patients (10, 38). We hope that by deeply studying the molecular mechanism of the pathogenesis of GI-NKTCL, looking for potential molecular targets and exploring new treatment methods, we can improve the prognosis of patients with GI-NKTCL.

Gene and pathway mutations may be related to the occurrence and development of GI-NKTCL, and as such, may also be indicative of potential targets for treatment. In this study, we found common driving mutations in the tumor suppressor gene TP53 (mainly in exons 5–8) (39), and oncogenes ARID1B, ERBB3, and POT1. TP53 acts as a tumor suppressor by inducing G1 cell cycle arrest in DNA damaged cells. Other functions of TP53 include the regulation of DNA repair, apoptosis, aging, and metabolism (4). In NKTCL, the mutation rate of TP53 is 20–60% (18), and is one of the potential reasons for the low survival rate of NKTCL patients (40). ErbB3 belongs to the ErbB family of receptor tyrosine kinases, and regulates the proliferation and survival of epithelial cells (41). It is highly expressed in common tumors such as breast cancer, melanoma, and pancreatic cancer (42–44), and when activated, can cause resistance to a range of anticancer drugs (45). One example is through the activation of PI3K/AKT and JAK/STAT signaling pathways, which has been shown to produce drug resistance in colon and non-small cell lung cancer patients (46–48). POT1 is an important gene that protects telomeres by inhibiting DNA damage and regulates telomere length *via* telomerase activity (49). Studies have shown that mutations in POT1 are often found in patients with B-cell lymphoma (50), and that POT1 gene mutation can lead to the occurrence of various tumors, such as lymphomas (51). Apart from these driving mutations, the gene with the highest mutation frequency in patients with GI-NKTCL was found to be the RETSAT gene(4/15, 29%). Research has indicated that this gene is important for promoting adipogenesis and normal adipocyte differentiation, and it follows that mutation of this gene may affect adipogenesis and differentiation of adipocytes (52). Many studies suggest that lipid metabolism plays a role in the occurrence and development of tumors, regulates cell proliferation (53) and invasion (54, 55), and influences the development of drug resistance (56, 57). Regarding pathway mutations in our study cohort, we found mutations in the G2M checkpoint pathway in 66.7% (10/15) of patients. The results from Song et al. are consistent with this, showing that the application of histone deacetylase (HDAC) inhibitor chidamide, and DNA damage agent etoposide to NKTCL cells not only played a synergistic role in anti-proliferation and enhanced apoptosis, but also made the cell cycle stop at the G2/M phase (58). Also of note, we found changes in TNFA signaling due to mutation in the NFKB pathway in 46.7% (7/15) of GI-NKTCL patients. This is consistent with the results of Zhong et al., who found that a relationship exists between an imbalance of TNFA receptor signal and the poor clinical characteristics of patients with diffuse large B-cell lymphoma (59).

There were some limitations in this study: 1) The number of GI-NKTCL samples included in this study was limited to 15 cases; and 2) No follow-up functional analysis of high-frequency gene mutations and signaling pathway mutations in GI-NKTCL was conducted in this study. 3) We were unable to validate the relationship between RETSAT and SNRNP70 and the prognosis of immunotherapy in patients in the GI-NKTCL cohort due to the lack of survival data for immunotherapy in the GI-NKTCL cohort. Furthermore, the relationship between RETSAT and SNRNP70 and the prognosis of immunotherapy in patients with different cancers may be different due to tumor heterogeneity among different cancer types. In the future, we hope to further validate this by collecting data from patients with multiple cancer types receiving immunotherapy.

## Conclusions

In this study, we discovered the most common mutant genes and pathway mutations in a cohort of GI-NKTCL patients by analyzing genome level data obtained *via* WES. The most common mutated genes in GI-NKTCL patients are RETSAT and SNRNP70. Additionally, in G2M checkpoint and E2F targets are the most commonly mutated signaling pathways. In-depth exploration of the genomic changes of GI-NKTCL is helpful in understanding the pathogenesis of this disease, and we hope that the results of this study can be beneficial for providing a theoretical basis for finding new biomarkers and therapeutic targets.

## Data availability statement

The original contributions presented in the study are included in the article/**Supplementary Materials**. Further inquiries can be directed to the corresponding authors.

## Ethics statement

The studies involving human participants were reviewed and approved by the Sixth Affiliated Hospital of Sun Yat-Sen University. The patients/participants provided their written informed consent to participate in this study.

## Author contributions

Conceptualization, YH and JX. Formal analysis, SL, TL, HL, and XZ. Visualization, SL, TL, HL, and XZ. Writing–original draft, TC, HY, WH, XL, and ML. Writing–review & editing, SL, TL, HL, XZ, TC, HY, WH, XL, ML, YH and JX. All authors read and approved the final manuscript.

## Funding

This work was supported by Guangzhou General Planned Project of Science and Technology (grant number 202102080283) and Key Project of Rural Science and Technology Commissioner of Guangdong Province (grant number KPT20190263).

## Acknowledgments

We thank the Genetron Health (Beijing) Co. Ltd.

## Conflict of interest

The authors declare that the research was conducted in the absence of any commercial or financial relationships that could be construed as a potential conflict of interest.

## Publisher’s note

All claims expressed in this article are solely those of the authors and do not necessarily represent those of their affiliated organizations, or those of the publisher, the editors and the reviewers. Any product that may be evaluated in this article, or claim that may be made by its manufacturer, is not guaranteed or endorsed by the publisher.

## Supplementary material

Supplementary Figure 1 Kaplan-Meier(KM) curve of GI-NKTCL patients.

Supplementary Figure 2 The mutation landscape of Li-NKTCL patients (top mutated genes frequencies ≥ 4).
