# Supplementary figures and images for: Integrated driver mutations profile of chinese gastrointestinal-natural killer/T-cell lymphoma
Source: Front Oncol. 2022 Aug 18;12:976762. doi: 10.3389/fonc.2022.976762 (PMC9434212; doi:10.3389/fonc.2022.976762)

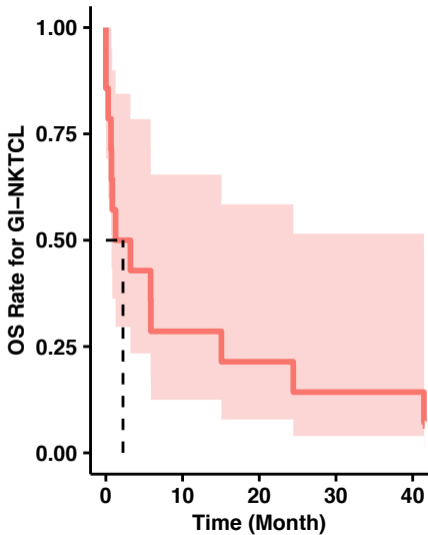

Supplement: Supplementary Figure 1 — Kaplan-Meier(KM) curve of GI-NKTCL patients. [file Image_1.pdf]

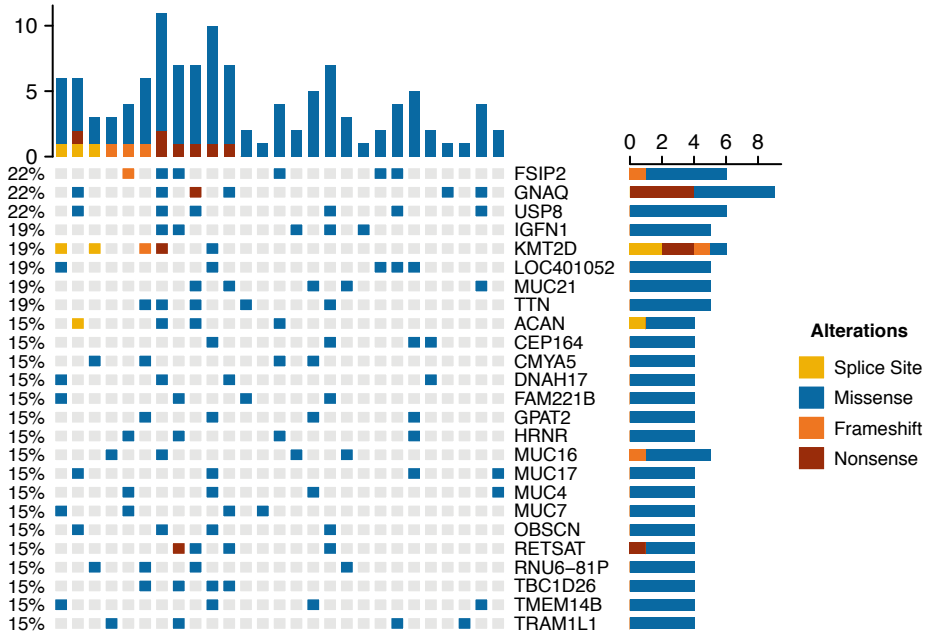

Supplement: Supplementary Figure 2 — The mutation landscape of Li-NKTCL patients (top mutated genes frequencies ≥ 4). [file Image_2.pdf]
