# Supplementary material for: Integrated driver mutations profile of chinese gastrointestinal-natural killer/T-cell lymphoma
Source: Front Oncol. 2022 Aug 18;12:976762. doi: 10.3389/fonc.2022.976762 (PMC9434212; doi:10.3389/fonc.2022.976762)
Supplement: Supplementary file 3 [file DataSheet_1.pdf]

## Supplementary Method

### Samples Collection

Tissue specimens (tumor tissues, matched non-tumor tissues or white blood cells) from gastrointestinal-Natural Killer/T-cell lymphoma (GI-NKTCL) patients with gastrointestinal lymphoma were collected with the informed consent of the patients. The study was approved by the Research Ethics Committee of the Sixth Affiliated Hospital of Sun Yat-sen University.

### DNA Extraction, Next Generation Sequencing Genomic

Genomic DNA was extracted from the fresh-frozen sample (or the Formalin‑fixed paraffin‑embedded (FFPF) sample) using a QIAamp DNA Mini kit (Qiagen, 51306, Valencia, CA, USA) according to the manufacturer's protocol. Agilent SureSelect XT Human All Exon v5 kit (Agilent Technologies, Santa Clara, CA, USA) was used to capture the exonic DNA fragments and a DNA library was constructed using KAPA Hyper Prep kits (KK8504, KAPA, Boston, MA, USA). Whole-exome of GI-NKTCL tumors and matched non-tumor tissues, or corresponding WBCs, were sequenced on the Illumina NovaSeq6000 platform.

### Bioinformatics Analysis

Data from tumor DNA were compared with DNA from matching normal tissue or blood cells ofthe same patient according to standardized computational workflow: Raw fastq files were trimmed and filtered by Trimmomatic 0.33 [1]. Filtered paired-end reads were aligned to the human reference genome (hg38) using the Burrows Wheeler Aligner (BWA, version 0.7.17-r1188) with defaults parameters [2]. Further duplicate detection, realignment, and reconstitution of the base-quality were performed with Picard tools (http://broadinstitute.github.io/picard/) and GATK [3]. Candidate somatic single nucleotide variations (SNVs) were called by using Mutect2 [4], and then COSMIC v95 and dbSNP150 as reference sets were used for known mutations of somatic and germline, respectively. By default, strelka (version 2.9.10) was used for small indels with BAM as input [5]. Variant Effect Predictor (VEP, version 106, parameters everything, fork 8, buffer_size 1000 ) was used for annotation of effects of variants [6].

All candidate somatic mutations were manually confirmed with the Integrative Genomics Viewer (IGV) (version 2.12.3) [7]. Four methods were used to identify significantly mutated genes (SMGs): MuSiC (v1.0.0; at least two FDR<=0.2) [8], MutSigCV (version 1.41; Benjamini-Hochberg false

discovery rate q value >0.1) [9], Oncodrive-FM (version 1.0.3; q-value <0.05) [10] and OncodriveCLUST (V1.0.0; q-value <0.05) [11]. Multiple software programs identified the gene as SMG. Tumor mutational burden (TMB) was defined as the number of somatic mutations in an individual's genome per megabyte (Mb).

# References


[1] Bolger AM, Lohse M, Usadel B. Trimmomatic: a flexible trimmer for Illumina sequence data. Bioinformatics. 2014. 30(15): 2114-20.

[2] Li H, Durbin R. Fast and accurate long-read alignment with Burrows-Wheeler transform. Bioinformatics. 2010. 26(5): 589-95.

[3] DePristo MA, Banks E, Poplin R, et al. A framework for variation discovery and genotyping using next-generation DNA sequencing data. Nat Genet. 2011. 43(5): 491-8.

[4] Cibulskis K, Lawrence MS, Carter SL, et al. Sensitive detection of somatic point mutations in impure and heterogeneous cancer samples. Nat Biotechnol. 2013. 31(3): 213-9.

[5] Kim S, Scheffler K, Halpern AL, et al. Strelka2: fast and accurate calling of germline and somatic variants. Nat Methods. 2018. 15(8): 591-594.

[6] McLaren W, Gil L, Hunt SE, et al. The Ensembl Variant Effect Predictor. Genome Biol. 2016. 17(1): 122.

[7] Robinson JT, Thorvaldsdóttir H, Wenger AM, Zehir A, Mesirov JP. Variant Review with the Integrative Genomics Viewer. Cancer Res. 2017. 77(21): e31-e34.

[8] Dees ND, Zhang Q, Kandoth C, et al. MuSiC: identifying mutational significance in cancer genomes. Genome Res. 2012. 22(8): 1589-98.

[9] Lawrence MS, Stojanov P, Polak P, et al. Mutational heterogeneity in cancer and the search for new cancer-associated genes. Nature. 2013. 499(7457): 214-218.

[10] Gonzalez-Perez A, Lopez-Bigas N. Functional impact bias reveals cancer drivers. Nucleic Acids Res. 2012. 40(21): e169.

[11] Tamborero D, Gonzalez-Perez A, Lopez-Bigas N. OncodriveCLUST: exploiting the positional clustering of somatic mutations to identify cancer genes. Bioinformatics. 2013. 29(18): 2238-44.
